# Measuring algorithmic bias to analyze the reliability of AI tools that predict depression risk using smartphone sensed-behavioral data

**DOI:** 10.21203/rs.3.rs-3044613/v1

**Published:** 2024-04-22

**Authors:** Daniel A. Adler, Caitlin A. Stamatis, Jonah Meyerhoff, David C. Mohr, Fei Wang, Gabriel J. Aranovich, Srijan Sen, Tanzeem Choudhury

## Abstract

AI tools intend to transform mental healthcare by providing remote estimates of depression risk using behavioral data collected by sensors embedded in smartphones. While these tools accurately predict elevated symptoms in small, homogenous populations, recent studies show that these tools are less accurate in larger, more diverse populations. In this work, we show that accuracy is reduced because sensed-behaviors are unreliable predictors of depression across individuals; specifically the sensed-behaviors that predict depression risk are inconsistent across demographic and socioeconomic subgroups. We first identified subgroups where a developed AI tool underperformed by measuring algorithmic bias, where subgroups with depression were incorrectly predicted to be at lower risk than healthier subgroups. We then found inconsistencies between sensed-behaviors predictive of depression across these subgroups. Our findings suggest that researchers developing AI tools predicting mental health from behavior should think critically about the generalizability of these tools, and consider tailored solutions for targeted populations.

from Click Therapeutics related to digital therapeutics. D.C.M has accepted honoraria and consulting fees from Boehringer-Ingelheim, Otsuka Pharmaceuticals, Optum Behavioral Health, Centerstone Research Institute, and the One Mind Foundation, royalties from Oxford Press, and has an ownership interest in Adaptive Health, Inc. J.M. has accepted consulting fees from Boehringer Ingelheim. G.J.A. holds equity in HealthRhythms, Inc. and Lyra Health, Inc., and has accepted consulting fees and honoraria from BetterUp and Quantum Health.

## Introduction

Mental healthcare systems are simultaneously facing a shortage of mental health specialty care providers and a large number of patients whose treatment needs remain unmet[1,2]. This service gap is driving research into AI-driven mental health monitoring tools, where sensed-behavioral data, defined as inferred behavioral data gathered by sensors and software embedded in everyday devices (e.g. smartphones, wearables), are repurposed to remotely monitor depression symptoms[3–7]. Sensed-behavioral data has also been referred to as personal, behavioral, or passive sensing data in other work[7]. AI tools that leverage sensed-behavioral data intend to near-continuously identify individuals experiencing elevated symptoms in-between clinical encounters and consequently deliver preventive care[8]. These tools can also be integrated into digital therapeutics to automate precision interventions[9]. Initial work showed that depression risk could be predicted from sensed-behavioral data at a similar accuracy to general practitioners[10] in small populations[5,11]. More recent work shows that these AI tools predict depression risk at an accuracy only slightly better than a coin flip in larger, more diverse samples[4,6,12,13]. This prior work has not specifically explored why accuracy is reduced in larger samples, and it is not clear how to improve AI tools for clinical use.

In this work, we hypothesized that accuracy is reduced in larger, more diverse populations because sensed-behaviors are unreliable predictors of depression risk: sensed-behaviors that predict depression are inconsistent across demographic and socioeconomic (SES) subgroups[14]. We intentionally use the term reliability due to its importance in both a psychometric and AI context. In a psychometric context, reliability refers to the consistency of a tool, typically a symptom assessment, across different contexts (e.g. raters, time)[14,15]. In AI, reliability is related to generalizability, if an AI tool is consistently accurate in different contexts (e.g. different populations, over time, etc.)[12]. Given these definitions, researchers in AI

fairness have argued that aspects of psychometric reliability are important in an AI context: similar inputs (e.g. sensed-behaviors) to an AI model should yield similar outputs (e.g. estimated depression risk)[16].

In this paper, we adapt these ideas to study a specific aspect of reliability important for mental health AI tools deployed in large populations, i.e. if similar sensed-behaviors are consistently related to depression risk across different groups of individuals. We hypothesize that if the sensed-behaviors predictive of depression risk are inconsistent across groups, AI models that use sensed-behaviors to predict depression risk will be inaccurate because similar sensed-behavioral patterns will indicate different levels of depression risk for different subgroups. For example, imagine that mobility positively correlates with depression risk in subgroup A, and negatively correlates with depression risk in subgroup B. An AI model trained across subgroups using exclusively mobility data, blind to subgroup information as is typically the case in this literature[3–5,17], will receive unreliable information – high mobility can simultaneously indicate both low and high depression risk – and will make incorrect predictions for one of the subgroups. We note upfront that in this manuscript we do not consider temporal aspects of reliability, though we acknowledge that this is important in discussions of psychometric reliability, specifically if the AI tool is consistently accurate for the same individual, with predictions made under similar conditions[18].

We tested this hypothesis by identifying population subgroups where a depression risk prediction tool underperformed, and then analyzed sensed-behavioral differences across these subgroups. We identified subgroups where the tool underperformed by measuring *algorithmic ranking bias* (hereafter referred to as "bias"), where individuals experiencing depression from one subgroup (e.g. older individuals) were incorrectly ranked by the tool to be at lower risk than healthier individuals from other subgroups (e.g. younger individuals)[19–22]. Reliability was analyzed by measuring ranking bias because if individuals in large populations have inconsistent relationships between sensed-behavior and mental health, behaviors that represent high depression risk for one subgroup may represent lower risk for another subgroup. For example, imagine an AI tool predicting that higher phone use increases depression risk. Studies[23,24] show that younger individuals have higher phone use than older individuals. Thus, the AI tool may incorrectly rank older individuals with depression to be at lower risk than healthier younger individuals, decreasing model accuracy (Fig. 1a).

Against this backdrop, we developed an AI tool that estimated depression symptom risk using behavioral data collected from individuals' smartphones, using similar sensed-behaviors and outcome measures from recent work[3–5,13,25] (Fig. 1b). The data used to develop and analyze the AI tool was collected during a U.S.-based National Institute of Mental Health (NIMH)-funded study[3,25–29], one of the largest, most geographically diverse studies of its kind. We then measured bias across attributes including age, sex at birth, race, household income, health insurance and employment to identify subgroups where the tool underperformed. We studied these specific attributes because of known behavioral differences across demographic and SES subgroups[23,24,30–32] that could impact the reliability of the developed AI tool. Finally, we interpreted why the tool underperformed by identifying inconsistencies between the AI tool and sensed-behaviors predicting depression across subgroups. A summary of this analysis can be found in Fig. 1.

## Results

### Data Collection

We analyzed data from a U.S.-based, NIMH-funded study conducted from 2019–2021 to identify associations between behavioral data collected from smartphones and depression symptoms[3,25–29]. Smartphone sensed-behavioral data on GPS location, phone usage (timestamp of screen unlock), and sleep were near-continuously collected from participants across the United States for 16 weeks and the PHQ-8, a self-reported measure of two week depression symptoms[33,34], frequently used in mental health research[3,5,25,27], was collected every three weeks beginning on the first week of the study (e.g. on weeks 1, 4, 7, …, known as weekly reporting periods). Sensed-behaviors were summarized over two weeks to align with collected PHQ-8 depression symptoms for prediction (see Table 1). For example, sensed-behaviors collected during weeks 3 and 4 were summarized to predict PHQ-8 values collected during week 4.

Table 1

**Sensed-behaviors.** An overview of the sensed-behavioral data used in this analysis. The same set of sensed-behaviors were collected from all participants, and were summarized over two week periods to align with self-reported PHQ-8 symptoms. Please see the methods for more details.

| Category | Derived Sensed-Behaviors |
|---|---|
| Location | Variance (variability in GPS location), number of unique locations, entropy (variability in unique locations), normalized entropy (entropy normalized by number of unique locations), duration of time spent at home, percentage of collected samples in-transition (participant moving at > 1 km/hour), and circadian movement (24-hour regularity in movement). Location sensed-behaviors were directly calculated over two week periods. For example, we calculated the number of unique locations over two weeks. |
| Phone usage | Duration of phone usage and number of screen unlocks each day and within four 6-hour periods (12-6AM, 6-12PM, 12-6PM, 6-12AM). The average and standard deviation of each phone usage sensed-behavior was calculated over two weeks, and the number of days with daily phone use and use within each 6-hour period was summed. |
| Sleep | Average sleep onset (beginning of sleep), average duration, and variability in duration over two weeks. |

Table 2 summarizes the data used for analysis. 3,900 samples were analyzed from 650 individuals, a large cohort and sample size compared to most studies to date analyzing associations between sensed-behaviors and mental health[4,5,25,35,36]. A sample was a set of sensed-behaviors, summarized over two weeks, with a corresponding PHQ-8 self-report. 46% of self-reported PHQ-8 values were ≥ 10, indicating clinically-significant depression (CSD)[33]. The majority of participants were relatively young to middle aged (75% 25 to 54 years old), female (74%), white (82%), middle to high income (61% annual household income ≥$40,000), insured (93%) and employed (62%). We focused our results on groups with at least 15

participants[37]. The sensed-behavior distributions across the population for each subgroup can be found in the supplementary materials.

Table 2

**Study cohort**. Data was collected within an NIMH-funded study to understand the relationships between digitally-collected behavioral data and depression symptoms[3,25–29]. Participants contributed six total samples (summarized behavioral data and depression outcome measures) throughout the course of the study. A sample was a set of sensed-behaviors, summarized over two weeks, with a corresponding PHQ-8 self-report.

| **Entire Study** | | | **Number of participants** | | **650** |
|---|---|---|---|---|---|
| | | | **Samples per participant** | | **6** |
| | | | **Total number of samples** | | **3,900** |
| | | | **% Clinically-significant depression** | | **46** |
| **Attribute** | **Group** | **Number of participants (%)** | **Attribute** | **Group** | **Number of participants (%)** |
| **Age** | 18 to 25 | 60 (9) | **Household Income** | < 20,000 | 98 (15) |
| | 25 to 34 | 181 (28) | | 20,000 to 39,999 | 144 (22) |
| | 35 to 44 | 168 (26) | | 40,000 to 59,999 | 124 (19) |
| | 45 to 54 | 135 (21) | | 60,000 to 99,999 | 161 (25) |
| | 55 to 64 | 81 (12) | | 100,000+ | 110 (17) |
| | 65 to 74 | 22 (3) | | Don't know | 10 (2) |
| | 75 to 84 | 3 (0) | | Prefer not to answer | 3 (0) |
| **Sex at Birth** | Female | 482 (74) | **Health Insurance Status** | Insured | 603 (93) |
| | Male | 168 (26) | | Uninsured | 43 (7) |
| **Race** | White | 534 (82) | | Don't know | 3 (0) |
| | Black/African American | 61 (9) | | Prefer not to answer | 1 (0) |
| | Asian/Asian American | 22 (3) | **Employment Status** | Employed | 401 (62) |
| | More than one race | 24 (4) | | Unemployed | 90 (14) |
| | Other | 6 (1) | | Disability | 72 (11) |
| | Prefer not to answer | 3 (0) | | Retired | 34 (5) |
| | | | | Other | 52 (8) |

| | | |
|---|---|---|
| **Entire Study** | **Number of participants** | **650** |
| | **Samples per participant** | **6** |
| | **Total number of samples** | **3,900** |
| | **% Clinically-significant depression** | **46** |
| | Prefer not to answer | 1 (0) |

## Measuring Bias to Identify Subgroups where AI Models Underperform

The PHQ-8 asked participants to self-report depression symptoms experienced over 14 days, and PHQ-8's were delivered multiple times throughout each weekly reporting period. We trained AI models using 14 days of smartphone sensed-behavioral data to predict if the average PHQ-8 value across each weekly reporting period (days 7 through 14, see Fig. 1c) indicated clinically-significant depression (CSD, PHQ-8 score ≥ 10[33]) symptoms. Surveys were delivered multiple times each reporting week, and individual surveys may only reflect "briefly" elevated symptoms (e.g. work stress on the day the survey was administered). For this reason, PHQ-8 values were averaged over each reporting week to predict a more stable estimate of self-reported symptoms. In addition, by predicting average PHQ-8 values instead of individual self-reports, we could make use of all available data while ensuring that samples did not overlap temporally. Model performance was assessed by performing 5-fold cross-validation, partitioning on subjects, and predictions across folds were concatenated to calculate model performance. To analyze performance variability due to specific cross-validation splits, we performed 100 cross-validation trials, shuffling participants into different folds during each trial.

AI models output a predicted risk score from 0–1 of experiencing CSD. We used the predicted risk to calculate common ranking bias metrics[20–22] (Fig. 2) across the subgroups in Table 2. These metrics were based upon the area under the receiver operating curve (AUC), which measured the probability models correctly predicted that CSD samples were ranked higher (in the predicted risk) than RH samples. We first calculated the AUC within each subgroup (the "Subgroup AUC"). Note that equal Subgroup AUCs do not guarantee high AUC across an entire sample. For example, Fig. 2a shows simulated data where an algorithm correctly predicted CSD risk within subgroups, but younger individuals, compared to older individuals, had higher predicted risk overall. Thus, healthy younger individuals were incorrectly predicted to be at higher risk than older individuals experiencing CSD. Two additional performance metrics assessed such errors. Specifically, the background-negative-subgroup-positive, or BNSP AUC (Fig. 2b) measured the probability that individuals experiencing CSD (the "positive" label) from a subgroup were correctly predicted to have higher risk than RH (the "negative label") individuals from other subgroups ("the background"), and the background-positive-subgroup-negative, or BPSN AUC (Fig. 2c), measured the probability RH individuals from a subgroup were correctly predicted to have lower risk than background individuals experiencing CSD.

The highest performing AI model (a random forest, 100 trees, max depth of 10, balanced class weights, see methods) achieved a median (95% confidence interval, CI) AUC of 0.55 (0.54 to 0.57) across trials. Note that this low AUC was expected: it is comparable to the cross-validation performance of similar depression symptom prediction tools developed in larger, more diverse populations[4,6,13], and motivates the objective of this work to study the reliability of these tools in larger populations.

Figure 3 shows the model results by each metric across subgroups. The Subgroup AUC was lower for males (median, 95% CI 0.52, 0.49 to 0.55), Black/African Americans (0.50, 0.46 to 0.54), individuals from low income households (<$20,000, 0.46, 0.43 to 0.50), uninsured (0.45, 0.41 to 0.51), and unemployed (0.46, 0.42 to 0.50) individuals, compared to the median subgroup AUC for each attribute (e.g. "Sex at Birth") across trials. The BNSP AUC increased with age (from 0.50, 0.46 to 0.52 for 18 to 25 year olds, to 0.67, 0.62 to 0.73 for 65 to 74 year olds), but decreased with household income (from 0.60, 0.58 to 0.63 for individuals from <$20,000 income households, to 0.45, 0.42 to 0.48 for individuals from $100,000 + income households). Individuals who were White (0.49, 0.46 to 0.52), male (0.52, 0.49 to 0.55), insured (0.47, 0.43 to 0.50), employed (0.43, 0.41 to 0.45), or identified with an "Other" type of employment (0.55, 0.52 to 0.59) also had lower BNSP AUC, compared to the median BNSP AUC for each attribute.

The BPSN AUC findings showed complementary trends: RH older individuals (e.g. 65 to 74, 0.46, 0.40 to 0.50), unemployed (0.38, 0.36 to 0.41), uninsured (0.47, 0.43 to 0.50), Black/African American (0.48, 0.45 to 0.50), females (0.52, 0.49 to 0.55), and individuals coming from lower income households (e.g. <$20,000 0.42, 0.39 to 0.44) had a lower BPSN AUC. Results were reasonably consistent across different types of models, within subgroup base rates (% samples with PHQ-8 $\geq$ 10) were sometimes, but not always, associated with the BNSP/BPSN AUC, and subgroup sample size did not appear to be associated with the Subgroup AUC (see supplementary materials).

## Isolating the Effects of Subgroup Membership on Model Underperformance

We wished to account for intersectional identities (e.g. female and employed) and isolate the effect of subgroup membership on model underperformance. For an ideal classifier, the predicted risk would be low for RH subgroups, and high for CSD subgroups. In addition, we would expect groups with higher base rates (% of samples with PHQ-8 $\geq$ 10) to have a higher average predicted risk. We thus modeled expected differences from groups with either the lowest (for RH) or highest (for CSD) average risk across trials. Generalized estimating equations (GEE, exchangeable correlation structure)[38], a type of linear regression, was used to estimate the average effect of subgroup membership on the predicted risk after controlling across all other attributes. GEE was used instead of linear regression to correct for the non-independence of repeated samples across trials[38].

The regression results can be found in Fig. 4. The individuals with the lowest average predicted risk who were not experiencing depression were 18 to 25 years old, male, White, had a household income of $100,000+, were insured, and employed. The predicted risk was expected to be higher (95% CI lower-bound > 0) for RH individuals who were older than 34 (e.g. for 65 to 74 year olds, mean, 95% confidence interval 0.02, 0.01 to 0.04), identified as Asian/Asian American (0.02, 0.01 to 0.03), Black/African American (0.01, 0.00 to 0.01), came from <$60,000 income households (e.g. for <$20,000, 0.02, 0.01 to 0.03), were unemployed (0.03, 0.03 to 0.04), and/or on disability (0.01, 0.00 to 0.02). For individuals who were experiencing CSD, models predicted the highest average risk for 65 to 74 year olds, Females, Asian/Asian Americans, individuals who came from households with incomes of $20,000 to $39,999, were insured, and/or retired. The predicted risk for individuals experiencing CSD was expected to be lower (95% CI upper-bound < 0) if individuals were 18 to 25 (–0.02, − 0.04 to − 0.01), male (–0.01, − 0.02 to − 0.00), Black/African American (–0.02, − 0.03 to − 0.00), more than one race (–0.02, − 0.03 to − 0.00), White (–0.02, − 0.03 to − 0.01), came from any household with an annual income <$20,000 or ≥$40,000 (e.g. $100,000+ − 0.03, − 0.03 to − 0.02), and/or were employed (–0.02, − 0.03 to − 0.01). Predicted risk distributions often overlapped across subgroups with higher or lower risk, though there were general trends across subgroups (e.g. the predicted risk increased with age and unemployment in RH individuals, and risk decreased with income level for both CSD and RH individuals, see Fig. 4 for more details).

## Interpreting Sensed-Behaviors across Subgroups where Models Underperformed

We hypothesized that models underperformed because sensed-behaviors predictive of CSD were inconsistent across subgroups. We thus conducted an analysis to understand differences between how AI tools predicted CSD risk and the different relationships between sensed-behaviors and CSD across subgroups. First, we retrained the AI model on the entire data, and used Shapley additive explanations (SHAP)[39] to interpret how the AI tool predicted CSD risk from sensed-behaviors. We then compared SHAP values with coefficients from explanatory logistic regression models estimating how subgroup membership affected the relationship between each sensed-behavior and depression.

We found different relationships between the SHAP values (Fig. 5a) and sensed-behaviors associated with CSD across subgroups (Fig. 5b, comparisons across each attribute and feature can be found in the supplementary materials). For example, the AI tool predicted that higher morning phone usage (6AM – 12PM) was generally associated with lower predicted depression risk. Higher morning phone usage decreased depression risk for 18 to 25 year olds (mean, 95% CI effect on depression, standardized units: − 0.77, − 1.07 to − 0.47), but increased risk for 65 to 74 year olds (0.60, 0.07 to 1.12). Younger individuals, overall, also had higher morning phone use (standardized median, 95% CI 18 to 25 year olds: 0.32, − 2.27 to 1.60) compared to older individuals (65 to 74 year olds: − 0.62, − 1.96 to 0.76).

Figure 5a also shows that specific mobility features, including the circadian movement (regularity in 24-hour movement), location entropy (regularity in travel to unique locations), and the percentage of collected GPS samples in transition (approximated speed > 1 km/hour) were often associated with lower predicted CSD risk. Circadian movement decreased CSD risk for employed individuals (–0.16, – 0.24 to – 0.07), but increased CSD risk for individuals who were on disability (0.44, 0.21 to 0.66). Circadian movement and location entropy also decreased depression risk for individuals from middle income ($60,000 to $99,999) households (circadian movement: – 0.21, – 0.35 and – 0.07; location entropy: – 0.34, – 0.48 to – 0.20), but increased risk for individuals from low income (<$20,000) households (circadian movement: 0.30, 0.09 to 0.51; location entropy: 0.35, 0.14 to 0.57). Finally, a higher percentage of GPS samples in transition decreased depression risk for insured individuals (–0.15, – 0.22 to – 0.08), but increased risk for uninsured individuals (0.32, 0.11 to 0.52).

## Discussion

In this study, we hypothesized that sensed-behaviors are unreliable measures of depression in larger populations, reducing the accuracy of AI tools that use sensed-behaviors to predict depression risk. To test this hypothesis, we developed an AI tool that predicted clinically-significant depression (CSD) from sensed-behaviors and measured algorithmic bias to identify specific age, race, sex at birth, and socioeconomic subgroups where the tool underperformed. We then found differences between SHAP values estimating how the AI tool predicted CSD from sensed-behaviors, and explanatory logistic regression models estimating the associations between sensed-behaviors and CSD across subgroups. In this discussion, we show how differences in sensed-behaviors across subgroups may explain the identified bias and AI underperformance in larger, more diverse populations.

Measuring bias showed that models predicted older, female, Black/African American, low income, unemployed, and individuals on disability were at higher risk of experiencing CSD (high BNSP, low BPSN AUC), and younger, male, White, high income, insured, and employed individuals were at lower risk of experiencing CSD (high BPSN, low BNSP AUC), independent of outcomes. Comparing SHAP values to explanatory logistic regression coefficients suggests why AI models incorrectly predicted depression risk. For example, our findings show that younger individuals had higher daytime phone usage than older individuals. Models predicted that higher daytime phone usage was associated with lower CSD risk (Fig. 5a), potentially explaining why younger individuals, overall, had lower predicted risk, and older adults had higher predicted risk (Fig. 3). Differences could be attributed to younger individuals using phones for entertainment and social activities that support well-being, while older individuals may prefer to use their phones for necessary communication or information gathering[23].

In another example, the model predicted that mobility, measured through circadian movement, location entropy, and GPS samples in transition, was associated with lower CSD risk (see Fig. 5). Prior work has identified a negative association between these same mobility features and CSD[5,25], suggesting that mobility decreases depression risk. While we found the expected negative associations across majority, higher SES ($60,000 to 99,999 household income, insured, and employed) subgroups, we found the opposite, positive association across less-represented lower SES (<$20,000 household income, on disability, uninsured) subgroups, potentially explaining the reduced model performance (lower Subgroup AUC) in these groups. There are many possible explanations for the identified differences in behavior.

First, underlying reasons to be mobile (e.g. navigating bureaucracy to receive government payments) may increase stress for individuals who are lower income and/or on disability[31], increasing depression risk. Second, the analyzed data was collected during the early-to-mid stages of the COVID-19 pandemic, when mobility for low SES essential workers may indicate work travel and increased COVID-19 risk, contributing to stress[32] and depression. These findings suggest that sensed-behaviors approximating phone use and mobility used to predict depression in prior work[3–6,25] do not reliably predict depression in larger populations because of subgroup differences.

While existing work developing similar AI tools has strived to achieve generalizability[4,40], our findings question this goal. Instead, it may be more practical to improve reliability by developing models for specific, targeted populations[41,42]. In addition, it may be helpful to train AI models using both sensed-behaviors and demographic information. In prior work and this study, AI models were trained using exclusively sensed-behavioral data[3–5,17]. However, prior work suggests that models may not be more predictive even with added demographic information[43]. This shows that additional methods are needed to clearly define subgroups, beyond demographics, with more homogenous relationships between sensed-behaviors and depression symptoms.

Another method to improve reliability is to develop personalized models, trained on participants’ data over time[6,44]. While personalization seems appealing, researchers should ensure that personalized predictions are meaningful. For example, we experimented with personalized models using a procedure suggested from prior work[44]. The model AUC improved (0.68) compared to the presented results (0.55), but we achieved a better AUC (> 0.80) by developing a naive model re-predicting participants’ first self-reported PHQ-8 value for all future outcomes. Given at least one participant self-report is often needed for personalization, models should show greater accuracy than these naive benchmarks.

Even if accuracy improves, models can still be biased[19,37], and it is important to consider the clinical and public health implications of using biased risk scores for depression screening. For example, more frequent exposure to stress[45] contributes to higher rates of depression in lower SES populations[46], but overestimating depression risk for healthy low SES individuals allocates mental health resources away from other individuals who need care. Similar issues persist for underestimating depression risk. For example, models predicted lower risk for males experiencing depression compared to healthier females (see Fig. 3). Males are less likely to seek treatment for their mental health than females[47], and AI tools underestimating male depression risk may further reduce the likelihood that males seek care. Uncovering these biases are important before algorithmic tools are used in clinical settings.

To reduce these harms, researchers can use methods described in this and other work[37] to identify subgroups where AI tools underperform by measuring bias. Resources could then be directed to develop new or retrain existing models for these subgroups. Simultaneously, clinical personnel using these tools can be trained to identify algorithmic bias and mitigate its effects[48]. In addition, depositing de-identified sensed-behavior and mental health outcomes data in research repositories could increase available data to analyze the reliability of AI tools[12]. Finally, our findings show the importance of developing AI tools using data from populations that have similar behavioral patterns to the populations where these tools will be deployed. More thorough reporting of model training data[49], and monitoring AI tools in "silent mode", in which predictions are made but not used for decision making[50], could prevent AI tools developed in dissimilar populations from causing harm.

Finally, it is important to consider how the choice to classify depression symptom severity influenced our results, specifically choosing to predict binarized PHQ-8 values instead of raw PHQ-8 scores. Predicting binarized symptom scores is a fairly common practice in both the depression prediction literature[3–5,17], as well as in the clinical AI literature, broadly[51,52]. This practice is motivated by an interest to use AI tools for near-continuous symptom monitoring, in which an action (e.g. follow-up by a care provider) is triggered at a specific elevated symptom threshold. This motivation may be difficult to realize if the field continues to use depression symptom scales as outcomes; as recent work shows, these symptom scales do not produce categorical response distributions, with a clear decision boundary distinguishing individuals experiencing versus not experiencing symptoms. Instead, responses tend to exist along a continuum[14]. It is also important to consider if subgroup differences affect the interpretation and self-reporting of depression symptom scales. Despite this consideration, prior work provides evidence that the PHQ-8 exhibits measurement invariance across demographic and socioeconomic groups[53,54]. Thus, it may be unlikely that the bias identified in this work was due to group differences in self-reporting symptoms, but our findings could be partially attributed to the mistreatment of depression symptom scales as categorical in nature.

This work had limitations. First, we analyzed data from a single study, though the studied cohort was larger in size, geographic representation, and timespan compared to prior work. In addition, the study cohort was majority White, employed, and female, though we did not find that sample size was associated with classification accuracy. Only inter-individual variability was considered, not intra-individual variability, and thus these findings do not extend to longitudinal monitoring contexts, where changes in sensed-behaviors may indicate changes in depression risk. In addition, data was only analyzed from participants who provided complete outcomes data (participants who reported at least one PHQ-8 value during each of the 6 weekly reporting periods). Data was exclusively collected from individuals who owned Android devices, only specific data types (GPS and phone usage) were analyzed. Only smartphone sensed-behaviors were analyzed, and data collected from other types of devices (e.g. wearables) were not analyzed. Finally, data collection took place from 2019 to 2021, when COVID-19 restrictions varied across the United States, which may influence our findings. Future work can examine if these results replicate over larger, more diverse cohorts, in both demographic and socioeconomic attributes, as well as the devices used for data collection. In addition, future work can explore if sensed-behaviors are reliable predictors of depression in longitudinal monitoring contexts, though recent work suggests that sensed-behaviors have low predictive power, even when used for longitudinal monitoring[25].

In conclusion, we present one method to assess the reliability of AI tools that use sensed-behaviors to predict depression risk. Specifically we measured ranking bias in a developed AI tool to identify subgroups where the tool underperformed, and then we interpreted why models underperformed by comparing the AI tool to sensed-behaviors predictive of depression across subgroups. Researchers and practitioners developing AI-driven mental health monitoring tools using behavioral data should think critically about whether these tools are likely to generalize, and consider developing tailored solutions that are well-validated in specific, targeted populations.

### Methods

## Cohort

In this work, we performed a secondary analysis of data collected during a U.S.-based National Institute of Mental Health (NIMH) funded study. The motivation for this study was to identify smartphone sensed-behavioral patterns predictive of major depressive disorder (MDD)[3,25–29]. Participants were recruited from across the United States using digital registries and online advertisements, intentionally oversampling for individuals experiencing depression. Eligible participants lived in the United States, could read/write English, and owned an Android smartphone and data plan. In addition, eligible participants with at least moderate depression symptom severity based upon the Patient Health Questionnaire-8 (PHQ-8) $\geq 10$ were oversampled to create a sample with elevated symptoms. Individuals were excluded from the study if they self-reported a diagnosis of bipolar disorder, any psychotic disorder, shared a smartphone with another individual, or were unwilling to share data. Eligible participants were asked to provide electronic informed consent after receiving a complete description of the study. Eligible participants had the option to not provide consent, and could withdraw from the study at any point.

Consented participants downloaded a study smartphone application[55] and completed a baseline assessment to self-report demographic and lifestyle information. The study application passively collected GPS location, sampled every 5 minutes, and smartphone interactions (screen unlock and time of unlock) for 16 weeks. Individuals completed depression symptom assessments every 3 weeks within the smartphone application (the PHQ-8[33,34]). Data collection took place from 2019–2021, and all study procedures were approved by the Northwestern University Institutional Review Board (study #STU00205316).

## Sensed-Behavioral Features

We calculated sensed-behavioral features from the collected smartphone data to predict depression risk. Following established methods from prior work[3,5,25], we calculated GPS mobility features including the location variance (variability in GPS), number of unique locations, location entropy (variability in unique locations), normalized location entropy (entropy normalized by number of unique locations), duration of time spent at home, percentage of collected samples in-transition (participant moving at > 1 km/hour), and circadian movement (24-hour regularity in movement)[5]. We also calculated phone usage features from the screen unlock data[40], including the duration of phone use and the number of screen unlocks each day and within four 6-hour periods (12-6AM, 6-12PM, 12-6PM, 6-12AM). Finally, we used a standard algorithm[40,56] to approximate daily sleep onset and duration from screen unlock data.

## Classifying Depression Symptoms

The PHQ-8 asks participants to self-report depression symptoms that occurred during the past two weeks. Symptoms are reported from 0 (not experiencing the symptom) to 3 (frequently experiencing the symptom). Scores are summed and thresholded to classify severity, where summed scores of 10 or greater indicate a higher likelihood of experiencing a clinically-significant depression[33]. We thus followed prior work[5,25] to calculate sensed-behavioral features in the two week period up to and including each weekly PHQ-8 reporting period. Behavioral features were input into machine learning models to predict clinically-significant symptoms (PHQ-8 $\geq$ 10).

## Data Preprocessing

Screen unlock and sleep features were summarized to align with the PHQ-8[40]. The average and standard deviation of each daily and 6-hour epoch feature were calculated across the two week prediction period, and the number of days with daily phone use and use within each 6-hour epoch were summed. GPS features were directly calculated over the two weeks. As recommended by Saeb et al.[5], skewed features were log-transformed. Missing data was filled using multivariate imputation[57] and then standardized (mean = 0, standard deviation = 1) based upon each training dataset prior to being input into predictive models.

## AI Model Training and Validation

We trained machine learning models commonly used to predict mental health status from smartphone behavioral data including regularized (L2-norm) logistic regression (LR)[3,5], support vector machines (SVM)[4,58], and tree-based ensemble models including random forest (RF) and gradient boosting trees (GBT)[3,40]. We varied the strength of the LR and SVM regularization parameter (0.01, 0.1, 1.0), used a radial basis function SVM kernel, varied class balancing weights in the RF and SVM (unbalanced/balanced), varied the number of ensemble tree estimators (10, 100), depth (3, 10, or until pure), and the GBT learning rate (0.01, 0.1, 1.0) and loss (deviance and exponential). Non-logistic prediction models were calibrated using Platt scaling to approximately match the predicted risk to the proportion of individuals experiencing clinically-significant symptoms at each risk level[59]. Logistic regression models, as shown in prior work[59], output calibrated probabilities. Models were implemented using the scikit-learn Python library[60].

Multiple PHQ-8 surveys were administered each weekly reporting period (e.g. week 1, 4, 7, etc.). Survey scores in each reporting week were averaged to remove overlap between sensor and outcomes data. Data was analyzed from study participants who self-reported at least one PHQ-8 during each reporting week, resulting in 6 predictions per participant. Data from all other participants were removed (288 participants removed, 31% of total) to focus this analysis towards algorithmic bias due to group differences rather than bias due to missing outcomes data.

### Declarations

## Competing Interests

D.A. and T.C. have submitted patent applications related to this work. T.C. is a co-founder and equity holder of HealthRhythms, Inc. and has received grants from Click Therapeutics related to digital therapeutics. D.C.M has accepted honoraria and consulting fees from Boehringer-Ingelheim, Otsuka Pharmaceuticals, Optum Behavioral Health, Centerstone Research Institute, and the One Mind Foundation, royalties from Oxford Press, and has an ownership interest in Adaptive Health, Inc. J.M. has accepted consulting fees from Boehringer Ingelheim. G.J.A. holds equity in HealthRhythms, Inc. and Lyra Health, Inc., and has accepted consulting fees and honoraria from BetterUp and Quantum Health.

## Author Contributions

D.A. conducted the analysis and wrote the draft manuscript. T.C., F.W., J.M., C.A.S., and D.C.M. provided supervisory support throughout the analysis. D.C.M. and J.M. were involved in data collection. All authors participated in drafting and revising the manuscript.

## Acknowledgements

D.A. is supported by the National Science Foundation Graduate Research Fellowship under Grant No. DGE-2139899, and a Digital Life Initiative Doctoral Fellowship. Any opinions, findings, and conclusions or recommendations expressed in this material are those of the authors and do not necessarily reflect the views of the funders. Data collection was supported by NIMH Grant No. R01MH111610 to D.C.M. J.M. is supported by K08MH128640. C.A.S. is supported by T32MH115882. Computing costs were funded by a Microsoft Azure Cloud Computing Grant through the Cornell Center for Data Science for Enterprise and Society, awarded to T.C. T.C. and F.W. were also supported by a multi-investigator seed grant awarded from the Cornell Academic Integration program.

## Data Availability

Sensed-behavioral data cannot be made publicly available due to potentially identifying information (e.g. GPS location) that may compromise participant privacy. De-identified self-reported data (the PHQ-8) will be made available through the NIMH Data Archive.

## Code Availability

A repository for all code used for analysis can be found at the following link: https://github.com/dadler6/reliability_depression_ml.

## References

1. Cai, A. *et al.* Trends In Mental Health Care Delivery By Psychiatrists And Nurse Practitioners In Medicare, 2011–19. Health Aff. (Millwood) 41, 1222–1230 (2022).
2. Mohr, D. C. et al. Banbury Forum Consensus Statement on the Path Forward for Digital Mental Health Treatment. Psychiatr. Serv. appi.ps.202000561 (2021) doi:10.1176/appi.ps.202000561.
3. Liu, T. *et al.* The relationship between text message sentiment and self-reported depression. J. Affect. Disord. 302, 7–14 (2022).
4. Xu, X. *et al.* GLOBEM: Cross-Dataset Generalization of Longitudinal Human Behavior Modeling. *Proc. ACM Interact. Mob. Wearable Ubiquitous Technol.* 6, 190:1-190:34 (2023).
5. Saeb, S. *et al.* Mobile Phone Sensor Correlates of Depressive Symptom Severity in Daily-Life Behavior: An Exploratory Study. J. Med. Internet Res. 17, (2015).
6. Meegahapola, L. *et al.* Generalization and Personalization of Mobile Sensing-Based Mood Inference Models: An Analysis of College Students in Eight Countries. *Proc. ACM Interact. Mob. Wearable Ubiquitous Technol.* 6, 176:1-176:32 (2023).
7. Mohr, D. C., Shilton, K. & Hotopf, M. Digital phenotyping, behavioral sensing, or personal sensing: names and transparency in the digital age. Npj Digit. Med. 3, 1–2 (2020).
8. Lee, E. E. et al. Artificial Intelligence for Mental Health Care: Clinical Applications, Barriers, Facilitators, and Artificial Wisdom. Biol. Psychiatry Cogn. Neurosci. Neuroimaging S245190222100046X (2021) doi:10.1016/j.bpsc.2021.02.001.
9. Frank, E. *et al.* Personalized digital intervention for depression based on social rhythm principles adds significantly to outpatient treatment. Front. Digit. Health 4, (2022).
10. Mitchell, A. J., Vaze, A. & Rao, S. Clinical diagnosis of depression in primary care: a meta-analysis. The Lancet 374, 609–619 (2009).
11. Wang, R. *et al.* Tracking Depression Dynamics in College Students Using Mobile Phone and Wearable Sensing. *Proc. ACM Interact. Mob. Wearable Ubiquitous Technol.* 2, 43:1–43:26 (2018).
12. Adler, D. A. *et al.* A call for open data to develop mental health digital biomarkers. BJPsych Open 8, (2022).
13. Müller, S. R., Chen, X. (Leslie), Peters, H., Chaintreau, A. & Matz, S. C. Depression predictions from GPS-based mobility do not generalize well to large demographically heterogeneous samples. Sci. Rep. 11, 14007 (2021).
14. Fried, E. I., Flake, J. K. & Robinaugh, D. J. Revisiting the theoretical and methodological foundations of depression measurement. Nat. Rev. Psychol. 1–11 (2022) doi:10.1038/s44159-022-00050-2.
15. Beck, A. T. Reliability of psychiatric diagnoses: 1. a critique of systematic studies. Am. J. Psychiatry 119, 210–216 (1962).

16. Jacobs, A. Z. & Wallach, H. Measurement and Fairness. Proc. 2021 ACM Conf. Fairness Account. Transpar. 375–385 (2021) doi:10.1145/3442188.3445901.
17. Jacobson, N. C., Weingarden, H. & Wilhelm, S. Digital biomarkers of mood disorders and symptom change. Npj Digit. Med. 2, 1–3 (2019).
18. Boateng, G. O., Neilands, T. B., Frongillo, E. A., Melgar-Quiñonez, H. R. & Young, S. L. Best Practices for Developing and Validating Scales for Health, Social, and Behavioral Research: A Primer. Front. Public Health 6, (2018).
19. Obermeyer, Z., Powers, B., Vogeli, C. & Mullainathan, S. Dissecting racial bias in an algorithm used to manage the health of populations. Science 366, 447–453 (2019).
20. Borkan, D., Dixon, L., Sorensen, J., Thain, N. & Vasserman, L. Nuanced Metrics for Measuring Unintended Bias with Real Data for Text Classification. Preprint at http://arxiv.org/abs/1903.04561 (2019).
21. Kallus, N. & Zhou, A. The Fairness of Risk Scores Beyond Classification: Bipartite Ranking and the XAUC Metric. in Advances in Neural Information Processing Systems vol. 32 (Curran Associates, Inc., 2019).
22. Vogel, R., Bellet, A. & Clémençon, S. Learning Fair Scoring Functions: Bipartite Ranking under ROC-based Fairness Constraints. in Proceedings of The 24th International Conference on Artificial Intelligence and Statistics 784–792 (PMLR, 2021).
23. Andone, I. et al. How age and gender affect smartphone usage. in Proceedings of the 2016 ACM International Joint Conference on Pervasive and Ubiquitous Computing: Adjunct 9–12 (ACM, 2016). doi:10.1145/2968219.2971451.
24. Horwood, S., Anglim, J. & Mallawaarachchi, S. R. Problematic smartphone use in a large nationally representative sample: Age, reporting biases, and technology concerns. Comput. Hum. Behav. 122, 106848 (2021).
25. Meyerhoff, J. *et al.* Evaluation of Changes in Depression, Anxiety, and Social Anxiety Using Smartphone Sensor Features: Longitudinal Cohort Study. J. Med. Internet Res. 23, e22844 (2021).
26. Mohr, D. C. LifeSense: Transforming Behavioral Assessment of Depression Using Personal Sensing Technology. https://reporter.nih.gov/search/N6YCr94ZvkOVUNu1i5HNaQ/project-details/9982127 (2017).
27. Stamatis, C. A. *et al.* Prospective associations of text-message-based sentiment with symptoms of depression, generalized anxiety, and social anxiety. Depress. Anxiety 39, 794–804 (2022).
28. Meyerhoff, J. *et al.* Analyzing text message linguistic features: Do people with depression communicate differently with their close and non-close contacts? Behav. Res. Ther. 166, 104342 (2023).
29. Stamatis, C. A. *et al.* The association of language style matching in text messages with mood and anxiety symptoms. Procedia Comput. Sci. 206, 151–161 (2022).
30. Greissl, S. *et al.* Is unemployment associated with inefficient sleep habits? A cohort study using objective sleep measurements. J. Sleep Res. 31, e13516 (2022).

31. Iezzoni, L. I., McCarthy, E. P., Davis, R. B. & Siebens, H. Mobility Difficulties Are Not Only a Problem of Old Age. J. Gen. Intern. Med. 16, 235–243 (2001).
32. Levy, B. L., Vachuska, K., Subramanian, S. V. & Sampson, R. J. Neighborhood socioeconomic inequality based on everyday mobility predicts COVID-19 infection in San Francisco, Seattle, and Wisconsin. Sci. Adv. 8, eabl3825 (2022).
33. Kroenke, K. *et al.* The PHQ-8 as a measure of current depression in the general population. J. Affect. Disord. 114, 163–173 (2009).
34. Wu, Y. *et al.* Equivalency of the diagnostic accuracy of the PHQ-8 and PHQ-9: a systematic review and individual participant data meta-analysis. Psychol. Med. 50, 1368–1380 (2020).
35. Opoku Asare, K. *et al.* Predicting Depression From Smartphone Behavioral Markers Using Machine Learning Methods, Hyperparameter Optimization, and Feature Importance Analysis: Exploratory Study. JMIR MHealth UHealth 9, e26540 (2021).
36. Corponi, F. et al. Automated mood disorder symptoms monitoring from multivariate time-series sensory data: Getting the full picture beyond a single number. 2023.03.25.23287744 Preprint at https://doi.org/10.1101/2023.03.25.23287744 (2023).
37. Seyyed-Kalantari, L., Zhang, H., McDermott, M. B. A., Chen, I. Y. & Ghassemi, M. Underdiagnosis bias of artificial intelligence algorithms applied to chest radiographs in under-served patient populations. Nat. Med. 27, 2176–2182 (2021).
38. Ballinger, G. A. Using Generalized Estimating Equations for Longitudinal Data Analysis. Organ. Res. Methods 7, 127–150 (2004).
39. Lundberg, S. M. & Lee, S.-I. A Unified Approach to Interpreting Model Predictions. in Advances in Neural Information Processing Systems vol. 30 (Curran Associates, Inc., 2017).
40. Adler, D. A., Wang, F., Mohr, D. C. & Choudhury, T. Machine learning for passive mental health symptom prediction: Generalization across different longitudinal mobile sensing studies. PLOS ONE 17, e0266516 (2022).
41. Sperrin, M., Riley, R. D., Collins, G. S. & Martin, G. P. Targeted validation: validating clinical prediction models in their intended population and setting. Diagn. Progn. Res. 6, 24 (2022).
42. Mitchell, M. et al. Model Cards for Model Reporting. ArXiv181003993 Cs (2019) doi:10.1145/3287560.3287596.
43. Pratap, A. *et al.* The accuracy of passive phone sensors in predicting daily mood. Depress. Anxiety 36, 72–81 (2019).
44. Wang, R. et al. CrossCheck: toward passive sensing and detection of mental health changes in people with schizophrenia. in Proceedings of the 2016 ACM International Joint Conference on Pervasive and Ubiquitous Computing - UbiComp ’16 886–897 (ACM Press, 2016). doi:10.1145/2971648.2971740.
45. Williams, D. R., Mohammed, S. A., Leavell, J. & Collins, C. Race, Socioeconomic Status and Health: Complexities, Ongoing Challenges and Research Opportunities. Ann. N. Y. Acad. Sci. 1186, 69–101 (2010).

46. Everson, S. A., Maty, S. C., Lynch, J. W. & Kaplan, G. A. Epidemiologic evidence for the relation between socioeconomic status and depression, obesity, and diabetes. J. Psychosom. Res. 53, 891–895 (2002).
47. Chatmon, B. N. Males and Mental Health Stigma. Am. J. Mens Health 14, 1557988320949322 (2020).
48. Rajkomar, A., Hardt, M., Howell, M. D., Corrado, G. & Chin, M. H. Ensuring Fairness in Machine Learning to Advance Health Equity. Ann. Intern. Med. 169, 866–872 (2018).
49. Gebru, T. et al. Datasheets for Datasets. ArXiv180309010 Cs (2020).
50. Wiens, J. *et al.* Do no harm: a roadmap for responsible machine learning for health care. Nat. Med. 25, 1337–1340 (2019).
51. Wong, A. et al. External Validation of a Widely Implemented Proprietary Sepsis Prediction Model in Hospitalized Patients. JAMA Intern. Med. (2021) doi:10.1001/jamainternmed.2021.2626.
52. Adams, R. et al. Prospective, multi-site study of patient outcomes after implementation of the TREWS machine learning-based early warning system for sepsis. Nat. Med. 1–6 (2022) doi:10.1038/s41591-022-01894-0.
53. Galenkamp, H., Stronks, K., Snijder, M. B. & Derks, E. M. Measurement invariance testing of the PHQ-9 in a multi-ethnic population in Europe: the HELIUS study. BMC Psychiatry 17, 349 (2017).
54. Villarreal-Zegarra, D., Copez-Lonzoy, A., Bernabé-Ortiz, A., Melendez-Torres, G. J. & Bazo-Alvarez, J. C. Valid group comparisons can be made with the Patient Health Questionnaire (PHQ-9): A measurement invariance study across groups by demographic characteristics. PLOS ONE 14, e0221717 (2019).
55. Audacious Software. Passive Data Kit. https://passivedatakit.org/.
56. Abdullah, S., Matthews, M., Murnane, E. L., Gay, G. & Choudhury, T. Towards circadian computing: 'early to bed and early to rise' makes some of us unhealthy and sleep deprived. in Proceedings of the 2014 ACM International Joint Conference on Pervasive and Ubiquitous Computing 673–684 (ACM, 2014). doi:10.1145/2632048.2632100.
57. Buuren, S. van & Groothuis-Oudshoorn, K. mice: Multivariate Imputation by Chained Equations in R. J. Stat. Softw. 45, 1–67 (2011).
58. Tseng, V. W.-S. *et al.* Using behavioral rhythms and multi-task learning to predict fine-grained symptoms of schizophrenia. Sci. Rep. 10, 15100 (2020).
59. Niculescu-Mizil, A. & Caruana, R. Predicting good probabilities with supervised learning. in Proceedings of the 22nd international conference on Machine learning - ICML '05 625–632 (ACM Press, 2005). doi:10.1145/1102351.1102430.
60. Pedregosa, F. et al. Scikit-learn: Machine Learning in Python. ArXiv12010490 Cs (2018).

## Figures

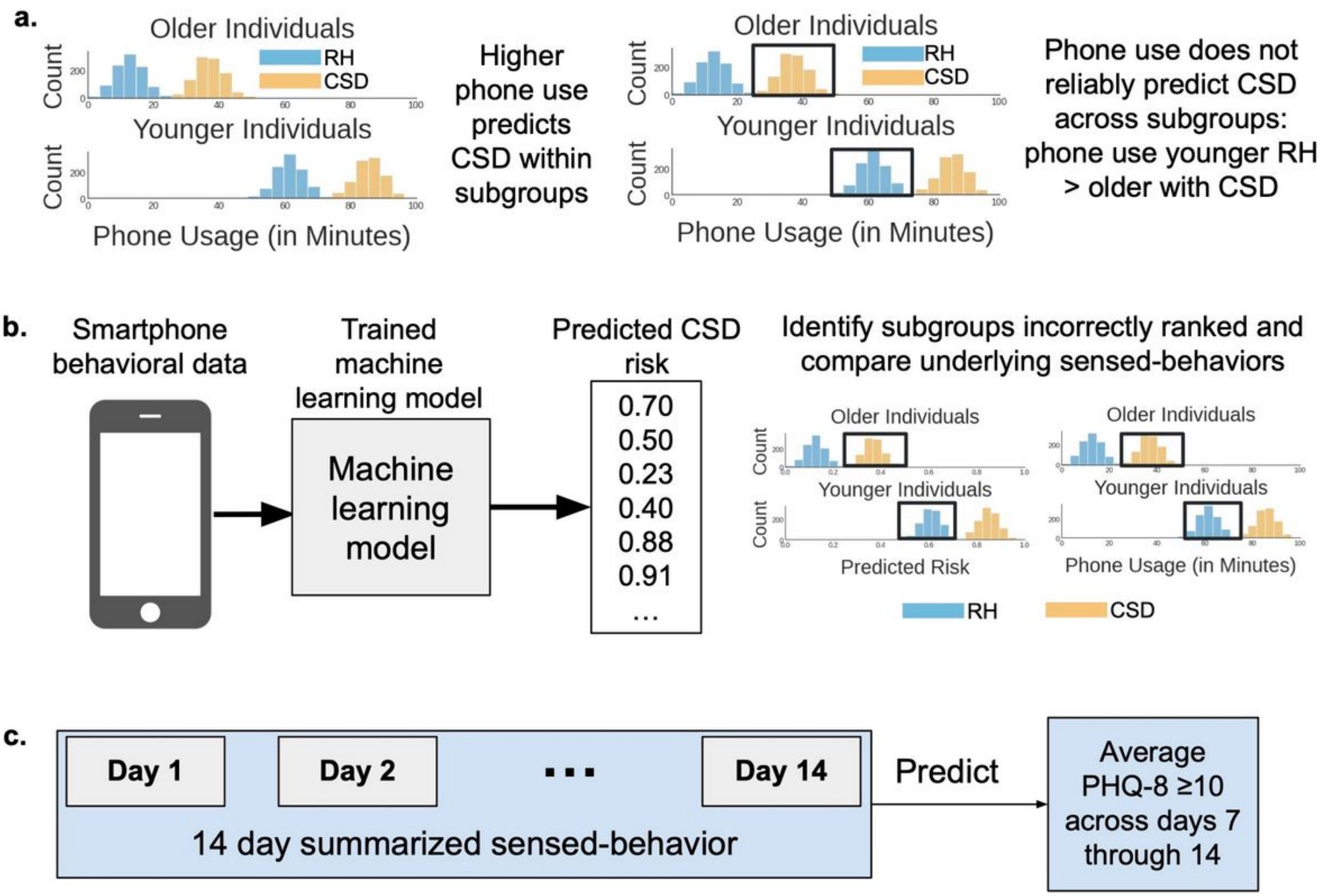

**Figure 1**

**Analyzing reliability in AI tools that predict depression symptom risk. a.** We hypothesized that sensed-behaviors, like phone use, unreliably predict depression in larger populations because behaviors representing high depression risk for some subgroups (e.g. older individuals) may represent lower risk for other subgroups (e.g. younger individuals). RH is relatively healthy and CSD is clinically-significant depression. Histograms show simulated data describing the count of individuals (y-axis) with specific daytime phone usage (x-axis). Colors indicate individuals experiencing CSD (orange) versus RH (light-blue). Plots are split by age subgroups. Black boxes show that increased phone usage is not a reliable predictor of depression because RH younger individuals have higher phone use than CSD older individuals. **b.** The analysis pipeline. Behavioral data from smartphones and mental health outcomes collected during a U.S.-based NIMH-funded study[3,25–29] were used to train and validate AI models that predicted depression symptom risk from the behavioral data. We then measured algorithmic ranking bias in the developed tool to identify subgroups where the predicted CSD risk was incorrectly ranked lower than RH subgroups, and compared sensed-behaviors across subgroups where algorithms underperformed. **c.** Similar to prior work[3,25], 14 days of sensed-behavioral data were used to predict whether the PHQ-8 value across each weekly reported period indicated clinically-significant depression symptoms (PHQ-8 ≥10[33]).

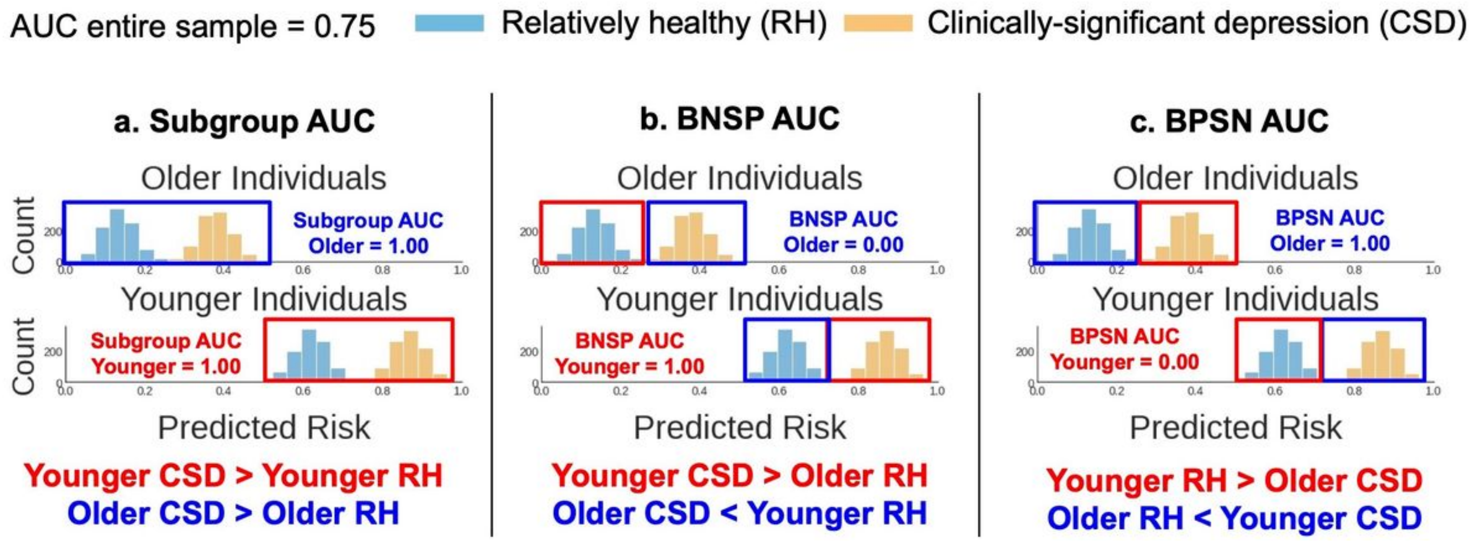

### Figure 2

**Measuring algorithmic ranking bias.** We considered three metrics from prior work to assess algorithmic ranking bias[20–22]. The predicted risk is the probability, output by the AI tool, that individuals were experiencing clinically-significant depression (CSD). Histograms show simulated example predictions from an AI tool, describing the count of individuals (y-axis) who fell into a predicted risk bin (x-axis). Colors indicate individuals experiencing CSD (orange) versus RH (light-blue). Plots are split by age subgroups (younger/older). The AUC is the area under the receiver operating curve. The red and dark-blue boxes, and corresponding text color below each plot, highlight the subgroups compared for each metric. **a.** The high Subgroup AUCs show that the predicted risk for individuals experiencing CSD was greater than the predicted risk for relatively healthy (RH) individuals within both age subgroups. But, this AI tool was biased to predict higher risk for younger individuals, overall, than older individuals. This bias is quantified using the **b.** Background-Negative-Subgroup-Positive (BNSP) AUC and **c.** Background-Positive-Subgroup-Negative (BPSN) AUC, which respectively show that younger individuals with CSD ("positive samples") were correctly ranked higher (high BNSP) than RH ("negative samples) samples from all other groups (older individuals, the "background"), but RH younger individuals were incorrectly ranked higher (low BPSN) than background samples with CSD. Older individuals show the complementary result (low BNSP, high BPSN). This bias reduces the model AUC when measured across the entire sample (assuming equal number of older and younger individuals, AUC=0.75), compared to the AUC in each subgroup (1.00).

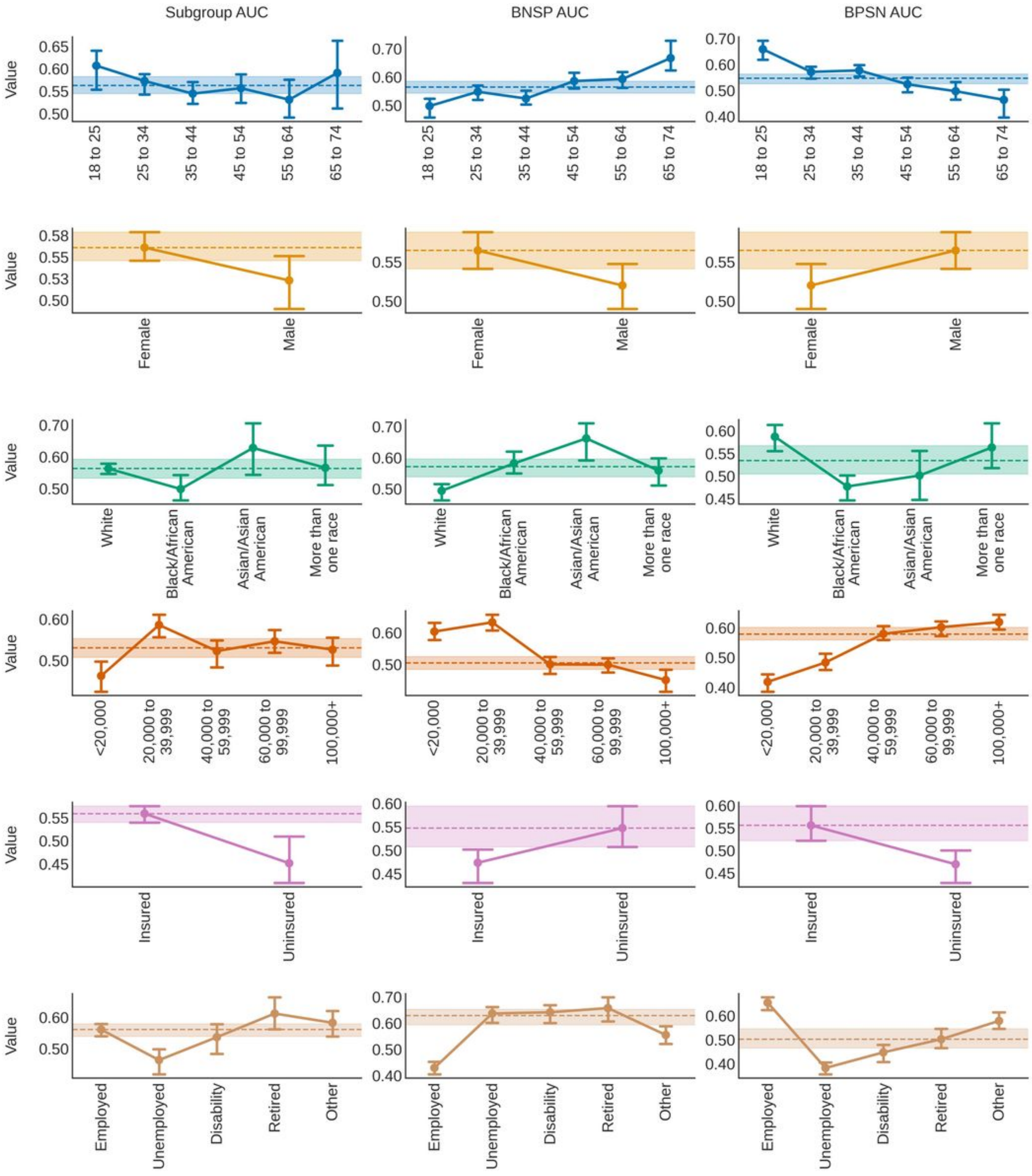

### Figure 3

**Measuring bias in predicted depression risk.** Bias was assessed by measuring the area under the receiver operating curve comparing positive (clinically-significant depression, CSD) and negative (relatively healthy, RH) samples within subgroups (Subgroup AUC, left column), subgroup positive samples to negative samples from all other groups, called "the background" (background-negative-subgroup-positive, or BNSP AUC, middle column), and subgroup negative samples to background positive samples

(background-positive-subgroup-negative, or BPSN AUC, right column)[20,22]. Point values indicate the median value across trials. Error bars show 95% confidence intervals (2.5 and 97.5 percentiles). Dotted lines and shaded areas show the distribution (median and 95% confidence intervals) of either the median (if >2 groups) or highest performing groups across trials.

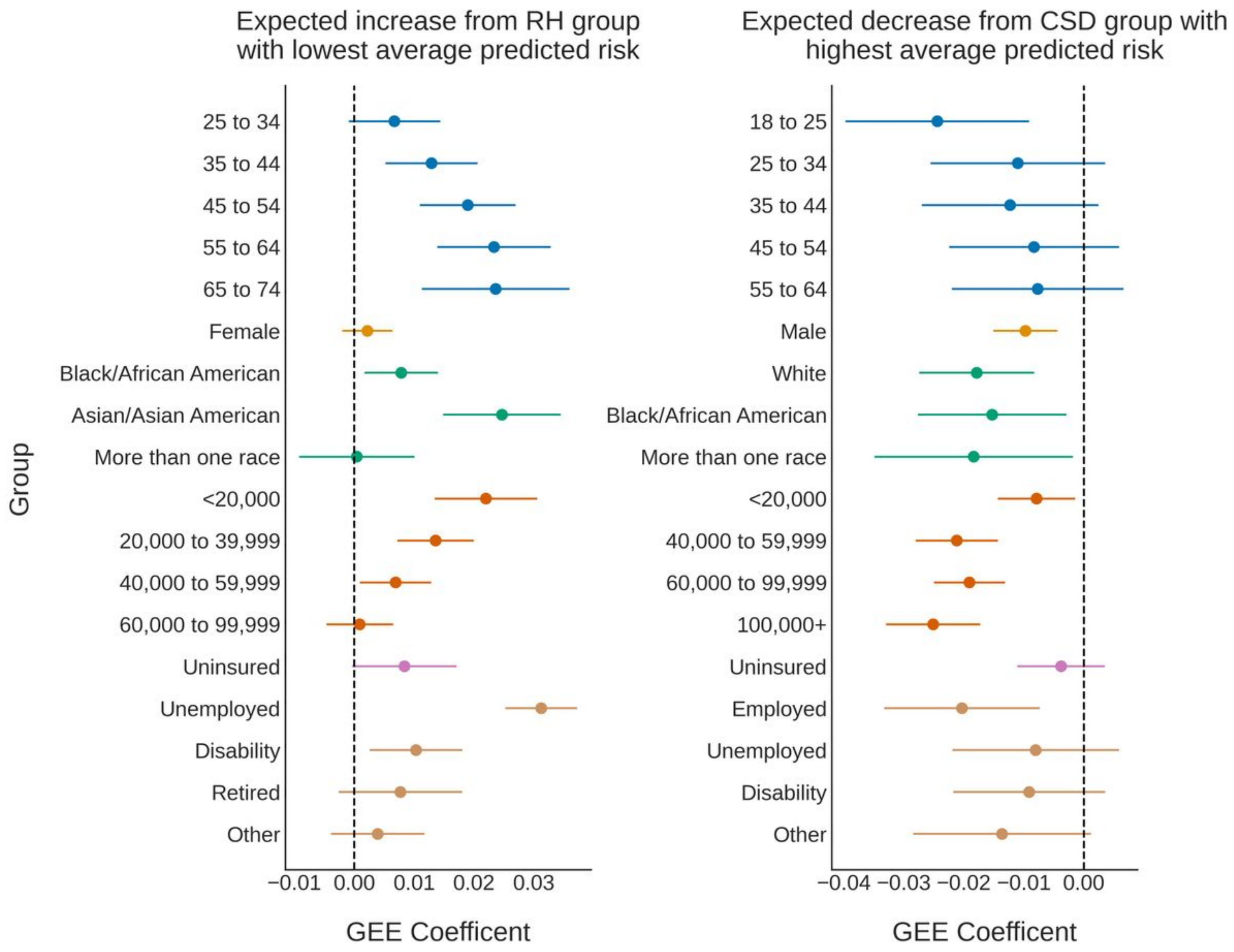

### Figure 4

**Isolating subgroups where models underperformed.** For an ideal classifier, the predicted risk would be low for relatively healthy (RH) individuals, and high for individuals with clinically-significant depression (CSD). We thus modeled expected differences from the subgroups with either the lowest (for RH, left) or highest (for CSD, right) average predicted risk across trials. Group effects were calculated using generalized estimating equations (GEE)[38], a type of linear model, to analyze the average effect of group membership on the predicted risk, controlling across all attributes. GEE accounted for the non-independence of repeated samples across trials[38]. Separate regression models were created for each outcome (RH, CSD) to remove the effects of the group base rate. Points represent the GEE coefficient

(expected effect), and error bars are 95% confidence intervals around the estimated effect. Dotted vertical lines highlight an expected group effect of 0.

a.

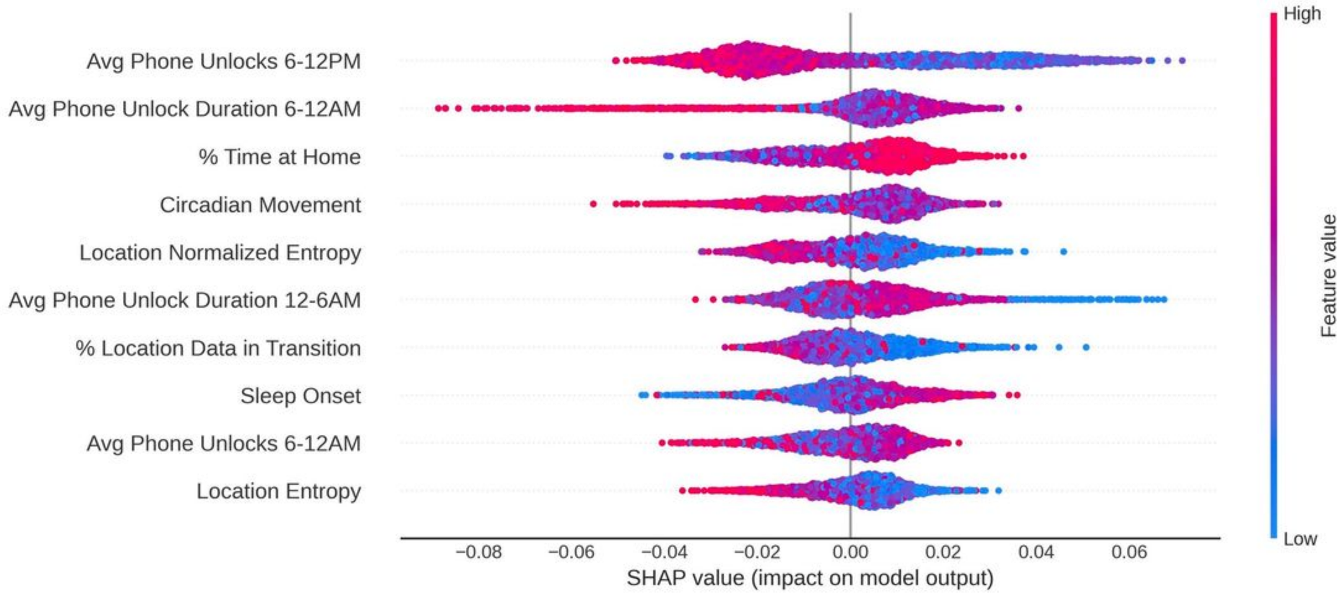

b.

| Sensed-Behavior | Subgroup | β (95% CI) | Median (95% CI) |
|---|---|---|---|
| Avg Phone Unlocks 6-12PM | 18 to 25 | −0.77 (−1.07 to −0.47) | 0.32 (−2.27 to 1.60) |
| Avg Phone Unlocks 6-12PM | 65 to 74 | 0.60 (0.07 to 1.12) | −0.62 (−1.96 to 0.76) |
| Circadian Movement | <20,000 | 0.30 (0.09 to 0.51) | −0.19 (−2.65 to 1.67) |
| Circadian Movement | 60,000 to 99,999 | −0.21 (−0.35 to −0.07) | 0.29 (−1.89 to 1.82) |
| Circadian Movement | Employed | −0.16 (−0.24 to −0.07) | 0.20 (−2.06 to 2.09) |
| Circadian Movement | Disability | 0.44 (0.21 to 0.66) | −0.20 (−2.34 to 1.53) |
| Location Entropy | <20,000 | 0.35 (0.14 to 0.57) | −0.37 (−1.21 to 2.87) |
| Location Entropy | 60,000 to 99,999 | −0.34 (−0.48 to −0.20) | 0.04 (−1.17 to 2.28) |
| % Location Data in Transition | Insured | −0.15 (−0.22 to -0.08) | −0.26 (−1.11 to 2.44) |
| % Location Data in Transition | Uninsured | 0.32 (0.11 to 0.52) | −0.16 (−1.10 to 4.44) |

### Figure 5

**Interpreting the relationships between sensed-behaviors and depression. a.** Shapley additive explanations (SHAP)[39] were used to interpret how the AI tool predicted depression risk using sensed-behaviors. Sensed-behaviors are ordered, descending, on the y-axis by their average impact on the predicted risk (the "SHAP value", x-axis). Only the top 10 sensed-behaviors with the highest average impact are listed, for space. Colors dictate whether a higher sensed-behavior "feature" value (red) is associated with higher or lower predicted risk. For example, higher average ("Avg") phone unlocks from 6-12PM was generally associated with lower predicted risk. Averages and deviations summarize sensed-behaviors over 14 days (see Figure 1c). **b.** Example coefficients (β, 95% CI, standardized units) from explanatory logistic regression models estimating the associations between sensed-behaviors and depression across subgroups, as well as the median and 95% CI of the sensed-behavior distribution. Full coefficients and statistics can be found in the supplementary materials.

## Supplementary Files

This is a list of supplementary files associated with this preprint. Click to download.

- SupplementaryWorkbookonSensedBehaviors.xlsx
- SupplementaryInformation.docx
- nreditorialpolicychecklist20230823.pdf
- nrreportingsummary20230823.pdf
